# Design of a testbed for mechanical and thermal stimulation in somatosensory studies

**DOI:** 10.1038/s41598-025-00026-1

**Published:** 2025-05-04

**Authors:** Marika Sperduti, Nevio Luigi Tagliamonte, Francesca Cordella, Loredana Zollo

**Affiliations:** Laboratory of Advanced Robotics and Human-Centred Technologies - CREO Lab, Universitá Campus Bio-Medico di Roma, Via Àlvaro Del Portillo 21, 00128 Rome, Italy

**Keywords:** Mechanical stimulation, Thermal stimulation, Somatosensory system, Stimulation testbed, Biomedical engineering, Chronic pain, Reflexes, Pain, Touch receptors

## Abstract

To address the low repeatability and accuracy of traditional technologies for testing the human somatosensory system, this work presents a novel mechatronic testbed. The testbed allows for the delivery of mechanical and thermal stimuli with a high spatial resolution, enabling continuous or discrete stimulation with a small fixed area and in a single experimental session. The testbed was employed to identify the mechanical/thermal innocuous and painful thresholds and the human ability to distinguish the nature of a painful stimulus, on both the hand and the forearm of 12 healthy volunteers. The results demonstrated the capability of the developed testbed to produce a range of forces that can induce different sensations (touch or pain). We found a statistical difference between the innocuous and painful thresholds, regardless of the tested anatomical spot. In this paper, a small thermal stimulation tip was appositely selected to study the reaction to a focused thermal stimulus that has been poorly investigated so far. The results highlighted a statistically significant difference between the two stimulated sites for the cool sensation and the hot pain. Moreover, the painful recognition task was sped up by the use of the developed testbed, which allowed a more fair comparison among the applied stimuli, increasing the accuracy, repeatability, and consistency when compared to the state-of-the art.

## Introduction

The somatosensory system has been widely studied over the years, but the underlying transduction mechanisms of mechanical and thermal stimuli are still not fully clarified. The identification of the mechanical^1–11^and thermal^1,6,12–16^thresholds have been the focus of multiple studies. Different devices have been used to induce mechanical sensation in humans, among which the Von Frey filament represents a gold standard for the mechanical threshold identification^1,3,6–8,10,12^. It was originally proposed for clinical evaluation of skin sensitivity to detect touch sensations^17^ and it consists of multiple nylon filaments with different diameters (Ø$~0.06-1$1 mm), each one with an associated force ($0.08-2940$mN). The filament is manually pressed perpendicularly to the tested surface until it starts to bend, which means the desired force is delivered^18^. These filaments are easy to use and versatile to evaluate the mechanical threshold in multiple anatomical sites. However, since they are manually applied, the delivered stimulus is highly dependent on to the experimenter so it is is barely repeatable, in terms of actual force, application velocity and position. Moreover, the applied stimulus can be only discrete and the stimulation area varies with the desired force (*i.e.*with the selected filament). In summary, the quality of the sensation perceived by the participant may be affected by the different dimensions of the probes^19^. To overcome these problems a hand-held pressure algometer is often employed^4,5,9,11,19,20^, even if this device is specifically designed to evaluate the pain thresholds. It is an electronic system with a fixed probe embedding a pressure-sensitive strain gauge at the tip and allows for the manual application of a continuous and measurable stimulus. It presents a fixed and larger area $(0.01-1$ cm$\phantom{0}^2$) when compared with the Von Frey filaments ($0.0032 - 1$ mm$\phantom{0}^{2}$), which has been demonstrated to have a lower effect on cutaneous afferent (specifically, when the area is greater than 0.5 mm$\phantom{0}^{2}$)^11^. Furthermore, the device is still operated manually, which limits its repeatability, as is the case of the Von Frey filament.

To increase the repeatability and resolution, an automated pressure algometer integrated into a positioning system, including a fixed ending tip with a large area ($10\times 10$ mm$\phantom{0}^{2}$), was developed in^11^. This device was specifically proposed to study pain thresholds only and has never been used to identify innocuous mechanical thresholds.

To overcome the limitations found in the presented available devices, a new testbed was developed in this work, which includes a mechanical stimulator accurately moved in a repeatable manner through a high-resolution 3-axis automated positioning system. The stimulator enables the application of a continuous stimulus using a small interchangeable tip (Ø$~0.3 - 3$ mm), allowing the application of the desired force with high resolution, constant area and controlled velocity. The stimulator can be either position- or force- controlled and both the actual force and position are continuously recorded. The possibility to deliver a continuous stimulation can increase the accuracy of the identified mechanical thresholds in comparison to the case of a discrete stimulation.

Human thermal stimulation is usually achieved through the use of a Peltier-based device^1,6,12–16^. It consists of a thermoelectric device able to produce both warm and cool stimuli, depending on the direction of the supply current, in a wide range of temperatures. The total area of the thermal stimulator ($5-24$ cm$\phantom{0}^2$) is usually fixed to the sample, and a ramp of temperature is applied, setting a range of $0-50^\circ$C.This range of temperature is usually selected since it can activate cold nociceptors (below $5~^\circ$C), cold receptors (below $25^\circ$C), warm receptors (above $36^\circ$C) and heat nociceptors (above $45^\circ$C)^21^. Thus, it allows the investigation and delivery of stimuli ranging from cold pain to hot pain.

Moreover, the thermal innocuous and painful thresholds are often identified by using the Marstock method or a modified version of it^1,6,14,15^. This method involves warming the device in contact with the skin from the baseline temperature of $32^\circ$C until a switch is pressed by the participant, which indicates the identified threshold. The temperature is then reversed until the cool threshold is identified with the same procedure. With this method, the optimal warming rate was identified as $1-1.5^\circ$C/s^22^. Whereas, in the modified Marstock method, once the threshold value has been identified, the temperature is returned to the baseline. The latter allows the investigation of the warm and the cool thresholds in two separate sessions^6^. In the testbed developed in this paper, we enclosed a thermal stimulator moved through the high-resolution positioning system mentioned above. It consists of a Peltier element connected to a custom aluminum tip with a small ending diameter ($\varnothing ~3$ mm). This small stimulation tip was specifically chosen to investigate the response to a focused thermal stimulus that has been poorly investigated so far. It allows the delivery of temperatures in the range $0-60^\circ$C,able to induce from cold pain to hot pain sensation, with an adjustable velocity.

Moreover, the human ability to discriminate the nature of a painful stimulus among mechanical, hot and cold has only been carried out once in the literature^23^. In this study the thermal stimulus was delivered through the use of a copper cylinder (with multiple areas $1-100$ mm$\phantom{0}^2$) pre-heated/cooled by immersing it in a hot/cold solution, producing a low repeatability and temperature accuracy. Instead, the mechanical stimulus was delivered by the use of a needle enclosed in a copper cylinder with a larger surface area. The stimuli were discrete and manually applied, thus limiting repeatability and accuracy.

The analysis of the literature reveals that the devices currently used for somatosensory studies have the following limitations: *i)* they typically do not integrate into a single platform a mechanical and thermal stimulator, making it more difficult to performe thermal and mechanical investigations in a single experimental session; *ii)* they can not produce focused thermal stimuli, which represents a limit in the investigation of punctual pathologies; *iii)* they are often manually applied, reducing the possibility of repeating accurately the stimulation at the same site; *iv)* in the case the application is automated, they are not suitable for investigating both touch and pain sensations.

To improve these aspects, which are fundamental for obtaining consistent and reliable results in somatosensory studies, this paper proposes the development of a novel testbed that is able to respond to the above mentioned needs not met by existing devices. More in detail, the presented testbed compared with the literature solutions enables: *i)* to enclose mechanical and thermal stimulators in a single setup, which would be a valuable addition allowing for multiple stimulations in a single experimental session and enabling a rigorous comparison of the results; *ii)* small thermal stimulation area, useful to evaluate the response of a specific anatomical site; *iii)* repeatability in the actual delivered force and in the application velocity, which allows a consistent comparison of the results from different experimental sessions; *iv)* high spatial resolution, to accurately stimulate the desired anatomical site; *v)* continuous mechanical stimulation associated with a fixed small stimulation area and continuous/discrete thermal stimulation, which makes the testbed highly versatile for multiple experiments.

The developed testbed could be useful for numerous studies, including the investigation of mechanical and thermal thresholds to restore physiological sensations in amputees using next-generation prosthetics with neuromorphic tactile feedback^24,25^, the testing of postoperative conditions, the exploration of mechanical innocuous and painful thresholds, the identification of chronic pain pathologies^2,3,26^.

The developed testbed was employed in a human somatosensory study described in detail in the following sections. In particular, it was applied to the human hand and forearm to identify the mechanical and thermal, innocuous and painful thresholds. Lastly, thanks to the integration of mechanical and thermal stimulators in a single device, the human ability to discriminate the nature of a painful stimulus among mechanical, hot and cold stimuli was also investigated.

**Fig. 1:**
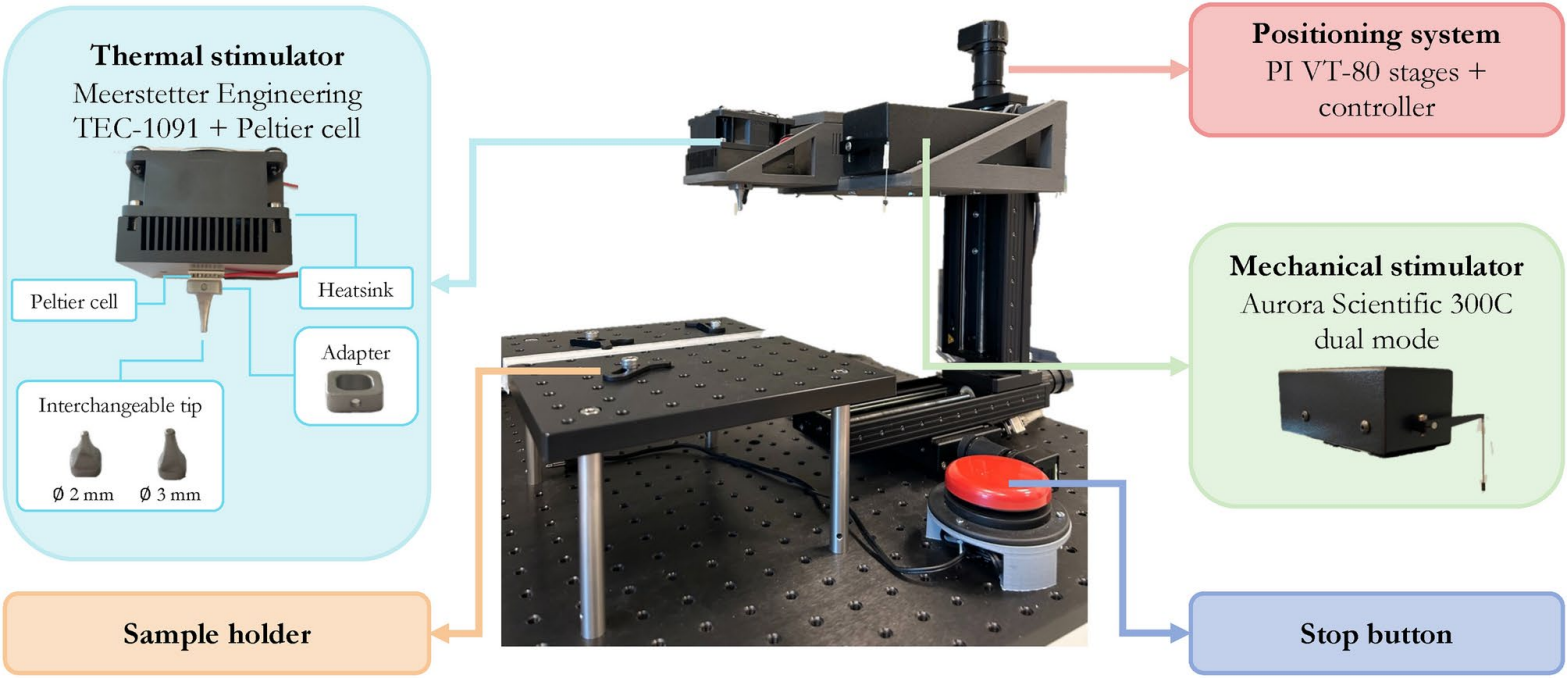
Experimental setup by means of: *i)* a positioning system, *ii)* a mechanical stimulator, *iii)* a thermal stimulator, *iv)* stop button, *v)* a positioning fixer.

## Materials and methods

### Testbed for mechanical and thermal stimulation

The developed mechatronic testbed, which was improved starting from the one described in^27^, is presented in this section with details of its components. The main technical specifications were defined starting from the analysis of the literature^28^, thus enabling the testbed to be used for a wide range of studies.

**Fig. 2:**
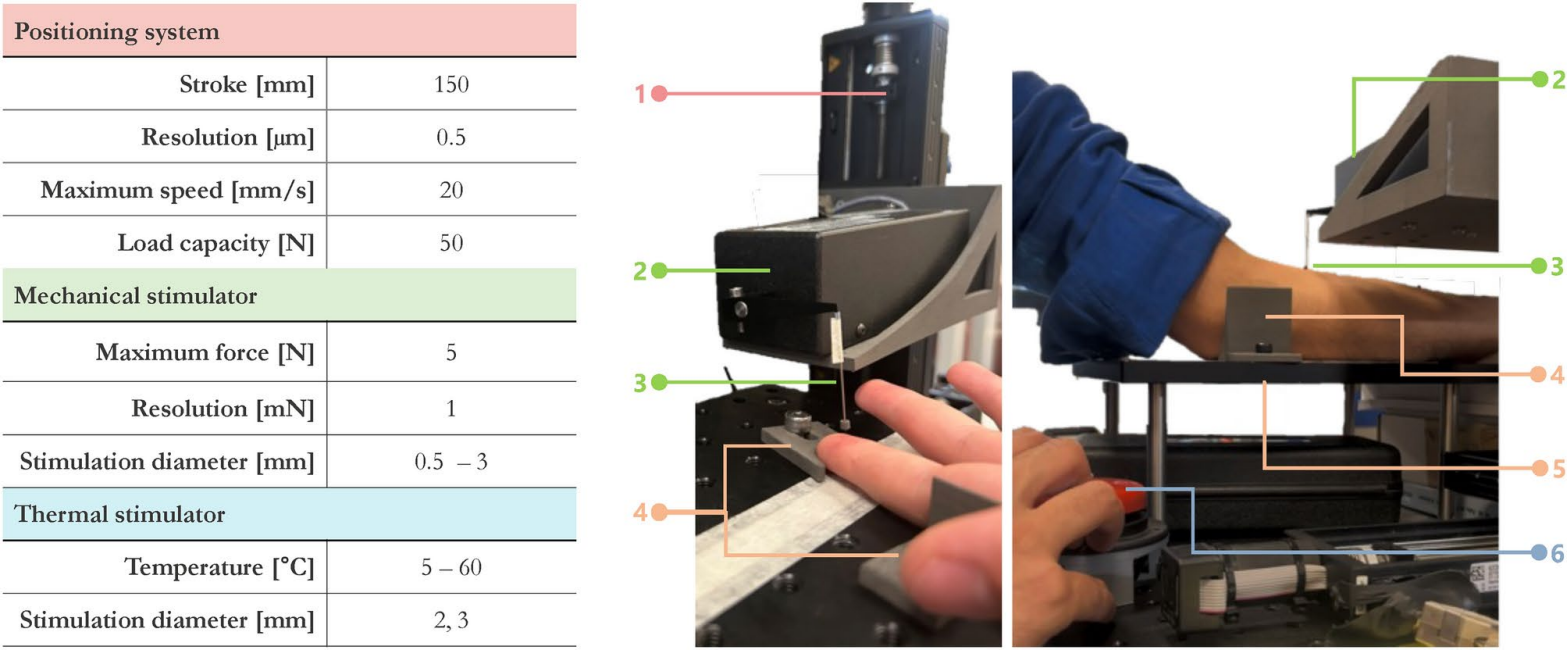
On the left the technical characteristics of: *i)* positioning system, *ii)* mechanical stimulator, *iii)* thermal stimulator. On the right details of the mechatronic testbed: over the fingertip on the left and over the forearm on the right. 1) Positioning system, 2) Mechanical stimulator: motor head, 3) Mechanical stimulator: stimulation tip, 4) Arm/hand restrainers, 5) Arm/hand holder, 6) Stop button.

The architecture and the main features of the system are reported in Figure 1 whereas the technical characteristics and details of its use are in Figure 2.

The testbed comprises both a mechanical and a thermal stimulator, alongside a high-resolution positioning system to increase the spatial repeatability. As a base frame, the Thorlab optical table MB6060/M (600$\times$600 mm$\phantom{0}^2$) was selected to include all the testbed modules. A detail description of each module is reported in the following sections.

#### Positioning system

The positioning system is made of a motorized 3-axis cartesian positioning system, which includes three linear stages VT-80 by Physik Instrumente, PI (Auburn, MA - United States) with DC motors, controlled by one multi-axis driver C-884.4DC by the same manufacturer, whose main features are reported in Figure 2. A 3D printed holder was used to integrate the vertical axis with mechanical and thermal stimulators, enabling both types of stimulation in a single experimental section.

#### Mechanical stimulator

The mechanical stimulator is based on the 300 C Dual Mode system device by Aurora Scientific (ON - Canada). This device was originally designed by the manufacturer to evaluate the mechanical properties of muscle and connective tissues on *in-vitro* and animal models and adapted in the present work for human stimulation. It is mainly composed of a motor head whose indenter ends with an interchangeable tip (ending diameter: 0.5 - 3 mm). In this paper the $\varnothing ~0.8$mm tip was used. The stimulator can be force- or position- controlled through an external analog signal and it can deliver a force of up to 5 N with a resolution of 1 mN, consistent with the range of force previously used in literature^4,5,9,19,20^. The system main characteristics are reported in Figure 2. Regardless of the selected control mode, both actual force and position are continuously recorded. To send analogue signals commanding the desired position or force, and also to acquire the actual delivered position and/or force analog signal, a DAQ from National Instruments, NI (USB-6212) (Texas - United Sates) was used and a custom user interface was developed by using NI Labview.

#### Thermal stimulator

A custom thermal stimulator was developed, by means of a Peltier cell (Adaptive, United Kingdom), with a surface of 15$\times$15 mm$\phantom{0}^{2}$, connected on one face to a heatsink, used to dissipate the heat produced when operating in cooling mode, and on the other face to a custom-made aluminum tip ($\varnothing$ 3 mm). The tip has been designed in a shape that allows the highest contact with the Peltier cell on one side and a circular ending on the other side. This device is also connected with two NTC (Negative Temperature Coefficient) thermal sensors: one is used as a feedback sensor to control the temperature of the tip, whereas the other one is used to monitor the temperature of the heatsink to avoid exceeding the maximum value allowed by the Peltier cell. The temperature regulation is performed by using a ThermoElectric Cooling (TEC) controller by Meerstetter Engineering (TEC-1091) (Rubigen, Switzerland). The developed thermal stimulator is able to control the temperature in the range 5- 60 $\phantom{0}^\circ$C, which is consistent with values used in literature^1,6,12,13,15,16,29–31^. The main characteristics of the thermal stimulator are reported in Figure 2.

#### User Interface

A stop button is integrated with the testbed to end the stimulation as soon as it is pressed and to record the instantaneously identified threshold. It communicates, via the NI DAQ, with the positioning system, the thermal and the mechanical stimulators. When it is pressed a trigger is recorded, the analog signal that controls the mechanical or thermal stimulator is reset and the positioning system is commanded to move to the homing position. Moreover, several arm/hand restrainers are designed and 3D printed to securely hold either the arm or the hand of the participant in a reproducible manner. They are used alongside a sample holder made of a small Thorlab optical table MB2025/M (200$\times$250 mm$\phantom{0}^2$) placed on 4 mounting posts (height: 100 mm).

#### Graphical user interface

To control the mechatronic testbed, a custom interface has been developed by using NI Labview. It allows the experimenter to set different stimulation profiles for the mechanical stimulator, also choosing between a force or position control of the indenter. Moreover, it enables to set the desired temperature as well as the heating/cooling velocity of the thermal stimulator. It is possible to move the single positioning axis, start and stop the stimulation, as well as define a stimulation pattern. This allows for multiple positions and different stimulations to be achieved automatically.

### Experimental protocol for thresholds identification

The somatosensory system and its underlying transduction principles have been the focus of multiple studies. The investigation of the mechanical and thermal thresholds is one of the main topics in this field. These studies are carried out with different aims, among which the assessment of pain sensitivity in healthy subjects^12,14^and in subjects affected by different patologies^6,12,15^, and the analysis of the effect of different stimulation sites on thresholds^13,30,32^represent leading aspects. With these aims multiple devices have been employed, each of which presents specific limitations, as deeply analized in^28^. In this paper, we have developed a new testbed that integrates a thermal and mechanical stimulator in a single device, which is designed to be applied in somatosensory studies with high accuracy and repeatability.

**Fig. 3:**
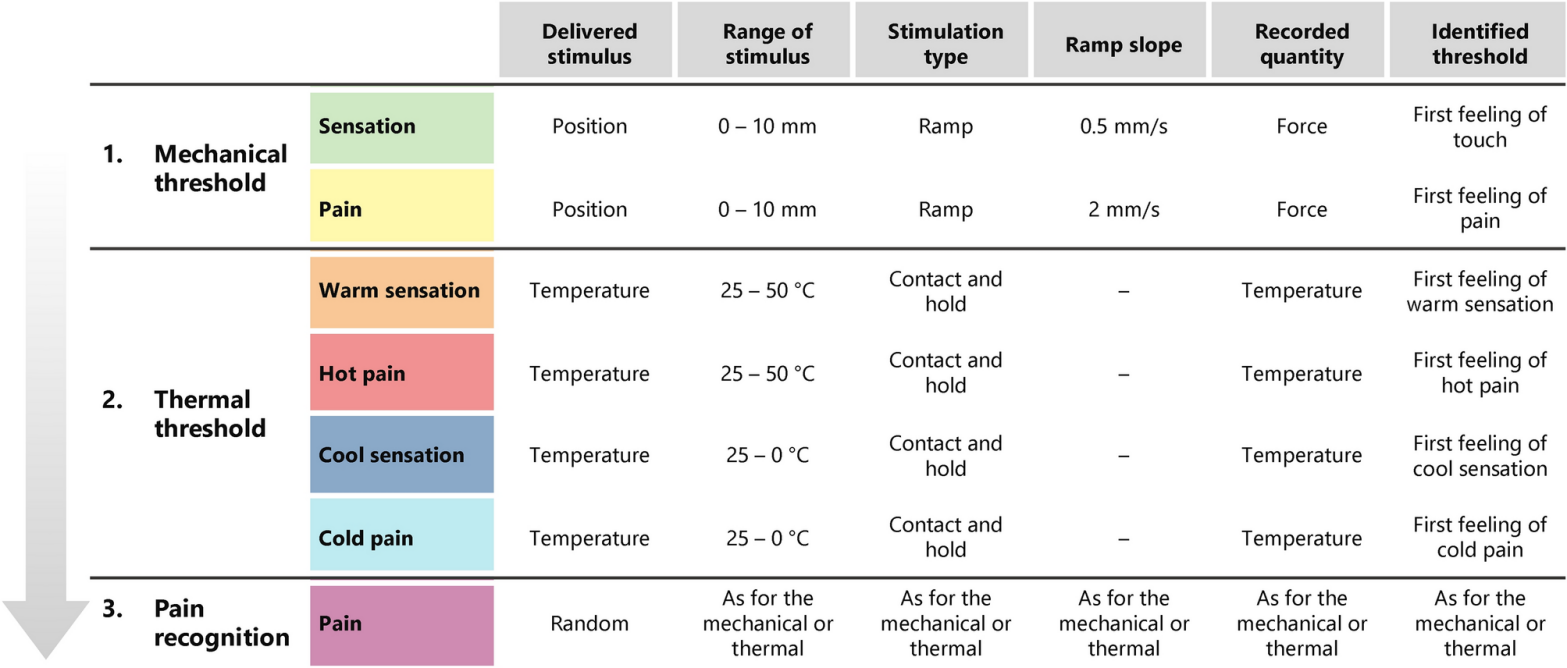
Detail of the specification used in the three successive phases of the protocol used.

In the experimental activities described in this section, 12 healthy volunteers (6 female and 6 male; age: 27.5 ± 2.2 y.o.) were recruited. The study was authorized by the Ethics Committee of Università Campus Bio-Medico di Roma (Territorial Ethics Committee Lazio Area 2: Prot. PAR: 79/21) in accordance with the Helsinki Declaration and following amendments; the main aspects of the study were explained to the participants in a comprehensive language and they signed an informed consent. The experiment was divided in three consecutive phases, namely mechanical threshold, thermal threshold and pain recognition. These phases are described more in detail in Figure 3.

Each participant was blinded and sitting comfortably in a chair. The index fingertip first and the inner part of the forearm later were positioned on the holder, fixed through the use of two 3D printed restrained as shown in Figure 2. The actual position of the mechanical and thermal tips over the desired stimulation spot was recorded before starting the experiments. An example of the mechanical stimulation over the two stimulated sites is represented in Figure 2. In this study the testbed was employed to examine the upper limbs. However, it can be easily adapted to other parts of the body (e.g., lower limbs) by adjusting the restraints and by checking the suitability of the desired force and temperature ranges.

#### Experimental identification of mechanical thresholds

First the Mechanical Innocuous Threshold (*MIT*) was investigated. The tip of the mechanical stimulator (in this study the tip $\varnothing ~0.8$ mm was used to deliver a focused stimulus) was positioned over the stimulation spot, setting the minimum distance in the vertical direction to guarantee that the subject did not feel any sensation due to the touch with the stimulator. Then the tip was position-controlled perpendicularly to the stimulated area by setting a velocity of 0.5 mm/s and setting a maximum excursion of 10 mm. To identify the MIT, the participant was asked to report the first touch sensation by pressing the stop button. As soon as the button was pressed, the testbed moved the mechanical stimulator away from the participant and both the position and force thresholds were recorded. The procedure was repeated three times. Afterward, to identify the Mechanical Pain Threshold (*MPT*), the same procedure was pursued by increasing the velocity to 2 mm/s. In this process, the participant was asked to report the first feeling of pain (*i.e.*the minimum sensation that induced discomfort) by pressing the stop button. As soon as the button was pressed, the testbed was moved away from the participant and both the position and force thresholds were recorded. Also this procedure was repeated three times. Preliminary results on the identification of mechanical thresholds were explored in^33^.

#### Experimental identification of thermal thresholds

First the Thermal Warm Sensation Threshold (*TWST*) was identified. The tip of the thermal stimulator (in this study the tip $\varnothing ~3$ mm was used to investigate a focused stimulus) was kept at room temperature and placed in contact with the hand so that the participant could feel only a touch sensation. Then, the tip was warmed with a velocity of 0.5 $\phantom{0}^\circ$C/s, by setting the maximum upper limit temperature at 60 $\phantom{0}^\circ$C. Since the mean reaction time for humans has been found to be 0.7–1.1 s on the fingertip and 0.4–1.1s on the arm^34^, and the minimum temperature variation detectable in humans is approximately 0.5 $\phantom{0}^\circ$C^35,36^, we decided to use a rate of 0.5 $\phantom{0}^\circ$C/s to ensure the proper detection of the temperature change.

The participant was asked to report the first warm feeling by pressing the stop button. As soon as the button was pressed the testbed moved the thermal stimulator away from the participant and the value of the temperature corresponding to the threshold was recorded. The procedure was repeated three times.

Afterward, to identify the Thermal Hot Pain Threshold (*THPT*), the same procedure was pursued, thus once that the tip at room temperature was in contact with the stimulated spot, it was warmed up to 60 $\phantom{0}^\circ$C with a speed of 0.5 $\phantom{0}^\circ$C/s. The participant was asked to report the first feeling of pain due to a hot stimulus by pressing the stop button. As soon as the button was pressed the testbed was moved away from the participant and the temperature corresponding to the hot pain threshold was recorded. The procedure was repeated three times.

The Thermal Cool Sensation Threshold (*TCST*) was then identified. The procedure is analogous to that employed for the TWST identification procedure. However, in this case, the tip was cooled, imposing a lower temperature limit at 0 $\phantom{0}^\circ$C. Furthermore, the participant was asked to report the first sensation of cool rather than warmth, by pressing the stop button.

Later on, the Thermal Cold Pain Threshold (*TCPT*) was identified. The procedure is analogous to that employed for the THPT identification procedure. However, in this case, the tip was cooled, imposing a lower temperature limit at 0 $\phantom{0}^\circ$C. Furthermore, the participant was asked to report the first sensation of pain due to a cold stimulus rather than painful hot, by pressing the stop button.

#### Experimental identification of pain nature

The capability of the participants to distinguish the nature of the applied painful stimulus was tested. The position and temperature associated with the mechanical and cold/hot pain, as reported from the previously described experiments, was applied to the hand randomly, with a total of 9 repetitions. More in detail, the mechanical stimuli were delivered by means of a ramp with a slope of 2 mm/s and a maximum displacement corresponding to the one identified as a painful threshold, whereas the thermal stimuli were delivered by laying the pre-heated/pre-cooled tip in contact with the participant for 1 s, having set the temperature as the one previously identified as a painful threshold. As soon as the stimulus was delivered, the participant was asked to recognize and verbally report the kind of felt stimulus, among mechanical, hot and cold pain. No feedback was given to the participant on the correctness of their answer until the end of the experiment.

The whole process was pursued first at the fingertip and then repeated at the forearm. The order of the two stimulated sites was arbitrarily selected.

### Extraction of literature data

To assess the suitability of the developed testbed for use in somatosensory studies, the experimental results were compared with reference data extracted from the literature. The consistency of our results with the identified studies was evaluated and the discrepancy with data obtained using different technologies was considered. In particular, the mean and standard deviation (when available) of the MIT and MPT on the hand and the forearm were extracted from relevant studies^4,5,9,19,20^. To ensure a correct comparison, the force data was converted into pressure. It is particularly important to highlight that in the analyzed studies the Von Frey filament (with a stimulation area of $0.005-0.09$ mm$\phantom{0}^2$) for the MIT and the algometer (with a stimulation area of $0.785-100$ mm$\phantom{0}^2$) for the MPT were used. Finally, the mean and standard deviation of the TWST, THPT, TCST, and TCPT (when available) were extracted from each analyzed paper and compared with the experimental results^1,6,12,13,15,16,29^. In the analyzed studies the stimulus was applied with a Peltier-based device. However, it should be noted that the stimulation area was significantly larger ($5-24$ cm$\phantom{0}^2$) than the one used with the developed testbed (0.07 cm$\phantom{0}^2$). A detailed examination of the data extracted from the literature can be found in section 3.

### Statistical analysis

A statistical analysis was performed to compare the two stimulated sites for all the applied stimuli, to investigate the possible difference in the perceived threshold depending on the stimulated spot. Moreover, a statistical analysis was performed (for each stimulated spot) to pairwise compare MIT and MPT, TWST and THPT, TCST and TCPT, with the aim to demonstrate the participant’s ability to discriminate between an innocuous and a painful stimulus. In this study, Wilcoxon’s non-parametric statistical test was used and the significance level *p* was set at 0.05. When multiple comparisons were required, the Bonferroni correction was applied. More in detail, a Bonferrori factor of 2 was used, since two comparisons were made on each dataset (e.g., the MIT on the hand was compared with the MIT on the forearm and with the MPT on the hand).

## Results

The results on the investigation of the human mechanical and thermal innocuous and painful thresholds on the two different anatomical sites are reported in this section.

**Fig. 4:**
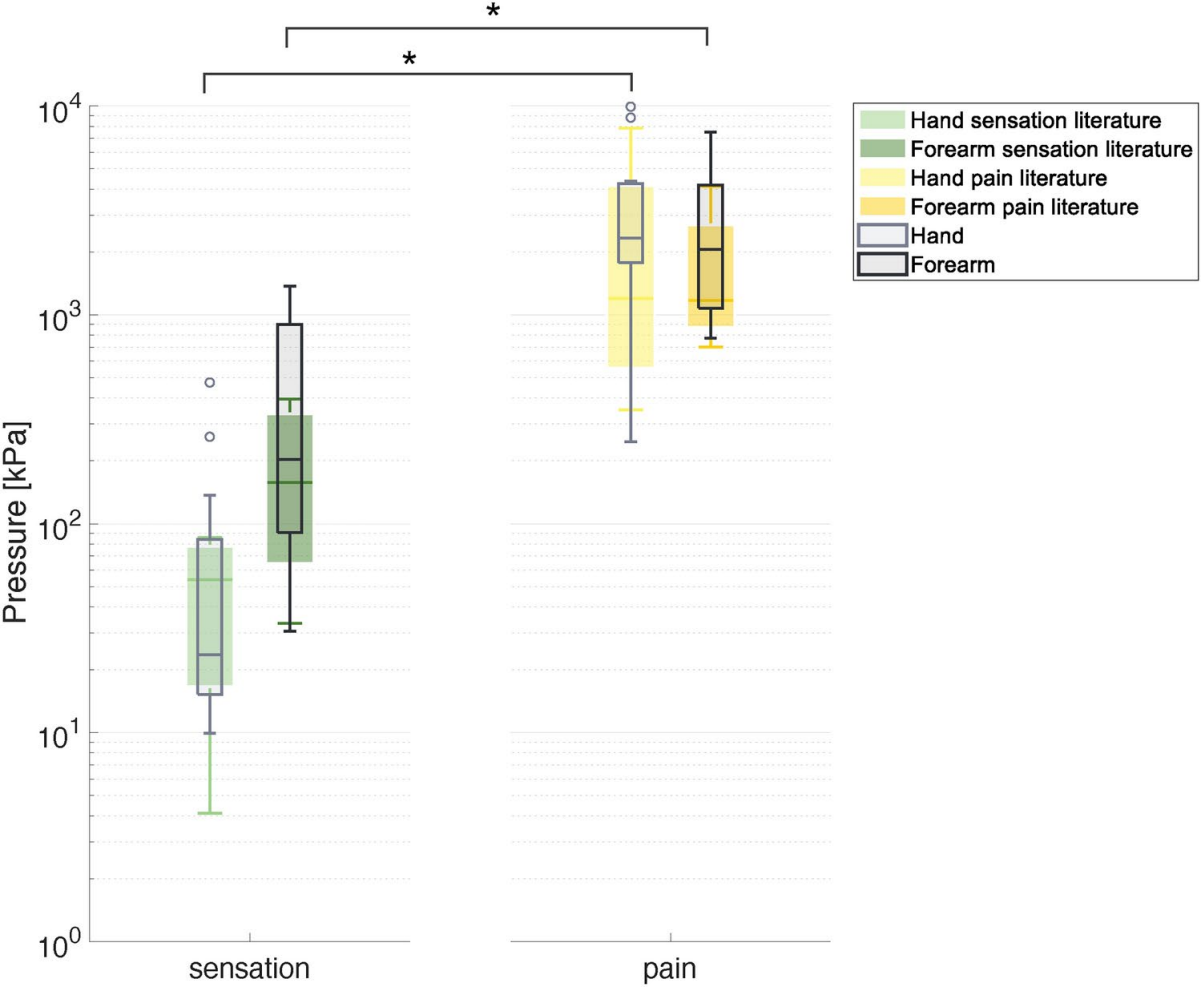
The box plot depicts the MIT and MPT in grey scale, while the data extracted from the literature are represented in colour. The data are reported in logarithmic scale. Asterisks indicate a statistically significant difference with $p\le 0.025$.

In Figure 4, the experimental results of the MIT and MPT are presented alongside the data extracted from the literature^4,5,9,19,20^. The results for the hand are depicted in grey, whilst those of the forearm are represented in black. Whereas, the data extracted from the literature are represented in light green for the MIT on the hand, in green for the MIT on the forearm, in light yellow for the MPT on the hand and yellow for the MPT on the forearm. Moreover, results from the statistical analysis are represented in Figure 4. Specifically, we compared the MIT and MPT between the hand and forearm, as well as the MIT and MPT on both stimulated sites. A more detailed comparison of our MPT results with the single literature studies is reported in Table 1. However, in these studies, the stimulation area is heterogeneous ($0.008-1.77$ cm$\phantom{0}^2$) and differs from the one used in our experimental setup (0.005 cm$\phantom{0}^2$). Moreover, the method used in the reviewed studies is based on an algometer, which requires the manual application of a controllable force in contrast with the automated stimulus applied with the developed testbed. It is worth highlighting that the results of the MIT cannot be directly compared with findings from available studies since two different technologies were used to deliver the stimulus: *i)* Von Frey filament with a discrete stimulus and a different contact area associated with each delivered force value, in literature data; *ii)* continuous stimulus with a fixed contact area, in this study.

The MIT and MPT experimentally identified in this paper are within the range of the literature data on both the hand and forearm. Moreover, consistently with previous studies, the MIT is higher on the forearm ($468\pm 499$ kPa) than on the hand ($88\pm 142$ kPa). Whereas, the MPT has a higher mean value on the hand ($3462\pm 2984$ kPa) when compared with the forearm ($2788\pm 2053$ kPa), as in the literature, with no statistically significant difference. A comparison of the MIT and the MPT reveals a statistically significant difference on both the stimulated sites (on the hand $p=5.98\,10^{-5}$ and on the forearm $p=4.78\,10^{-4}$).

**Fig. 5:**
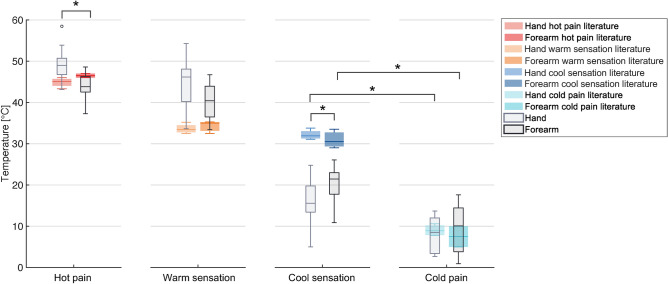
The identified temperature thresholds are represented in a grey scale, whereas data extracted from the literature are coloured. Asterisks indicate a statistically significant difference with $p\le 0.025$.

The same analysis, reported in Figure 5, has been performed for the thermal threshold identification. The experimental findings on the hand are reported in grey whereas the ones on the forearm are in black. Moreover, the data extracted from the literature are represented in different colors (with a lighter shade in the hand and a darker one in the forearm): red for the THPT, orange for the TWST, blue for the TCST, and light blue for the TCPT. The results from the statistical analysis are graphically represented in Figure 5 and are also summarized and compared with the data extracted from literature in Table 1^1,6,12,13,15,16,29^. It is important to note that in the analyzed studies, the used technology is Peltier-based as the one employed in our testbed, instead the stimulation area is considerably larger ($5-24$ cm$\phantom{0}^2$) than that used in the developed testbed (0.07 cm$\phantom{0}^2$).

The mean value of the experimental THPT on the hand ($49.3\pm 4.1~^\circ$C) is slightly higher than the literature data, but still comparable. The experimental results for the THPT on the forearm ($43.9\pm 3.5~^\circ$C) are slightly lower than the literature findings. Moreover, the experimental results for the THPT present a statistically significant difference when comparing the hand and the forearm ($p=0.005$). The TWST on both the hand ($44.7\pm 6~^\circ$C) and forearm ($40.2\pm 4.2~^\circ$C) are higher than in the literature. Moreover, the TCST both on the hand ($15.6\pm 5.9~^\circ$C) and on the forearm ($20.5\pm 4.4~^\circ$C), are lower than those extracted from the literature and showed a statistically significant difference when comparing the values on the hand and the forearm ($p=0.023$). Lastly, the TCPT on the hand and on the forearm ($7.9\pm 4.2~^\circ$C and $9.5\pm 6.2~^\circ$C, respectively) are within the range of the literature. Moreover, a statistically significant difference was found between the TCST and TCPT on the hand ($p=0.0017$), and on the forearm ($p=3.8\,10^{-4}$).

**Table 1:**
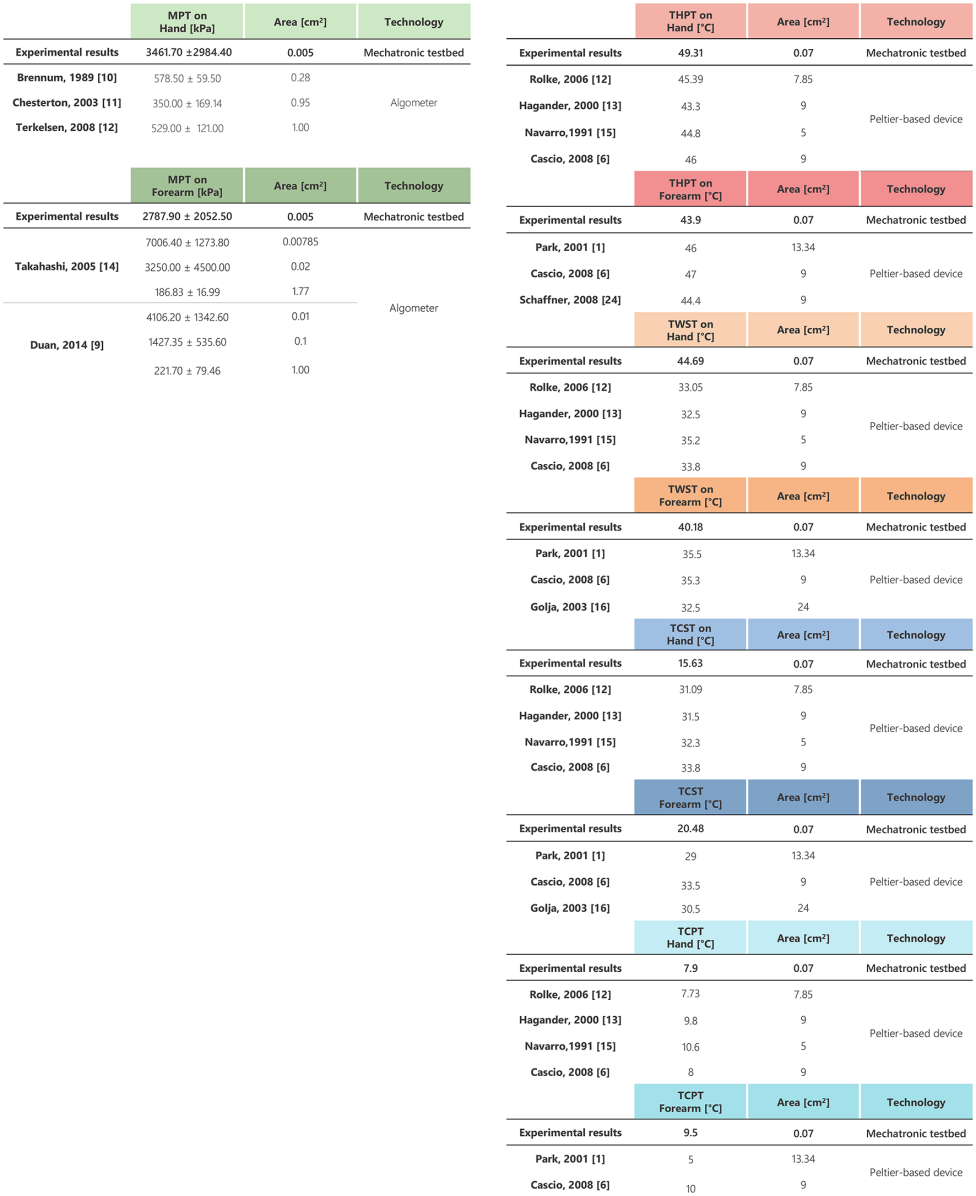
On the left, a comparison of data extracted from the literature for the MPT on the hand and forearm with the experimental results. On the right, a comparison of data extracted from the literature for the THPT, TWST, TCST and TCPT on the hand and forearm with the experimental results.

**Fig. 6:**
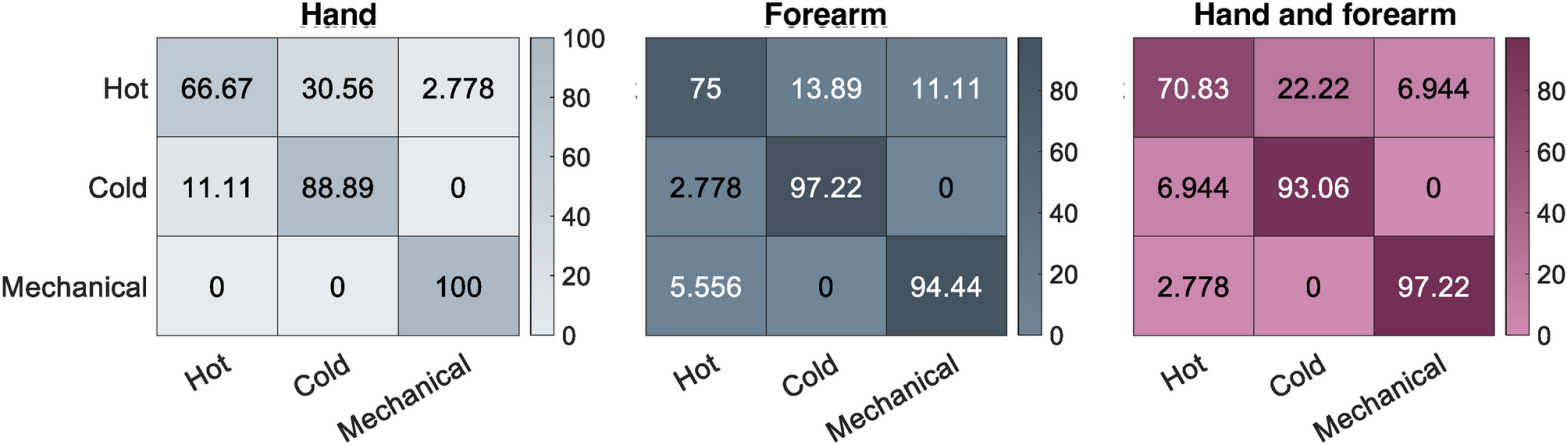
Recognition rate of the correct identifications of the nature of a painful stimulus.

Results on the ability to distinguish the nature of painful stimuli are reported in Figure 6. The data are presented in three confusion matrices: grey, black and purple for the hand, forearm and total results, respectively. The application of painful stimuli to the hand demonstrates that mechanical stimuli are the most easily distinguishable and consistently recognized (showing 100% of correct recognition rate). Whereas, in 11.1% of the cases, a cold stimulus is confused with a hot stimulus. The hot stimulus has proven to be the most difficult to identify. It is confused with the cold one in 30.6% of the cases and with the mechanical one in 2.8% of the cases. The results of the painful stimuli applied on the forearm show a similar trend to the results on the hand. However, the mechanical stimulus is confused with the hot one in 5.6% of the cases. Moreover, the cold stimulus is better recognized if compared with the cold stimulus applied on the hand, showing a confusion rate of 2.8% with a hot stimulus. The recognition rate of the hot stimulus is also increased to 75%. The final results of the hand and forearm are summarized in the confusion matrix reported in purple in Figure 6. The mechanical stimulus is the most widely recognized. Moreover, the cold stimulus is never confused with the mechanical one. Lastly, the hot stimulus is frequently mistaken for both the cold and mechanical stimulus. To investigate the inter-subject variability, the standard deviation on the correct answers among the subjects was evaluated. The results on the hand are: 66.7% ± 31.8 % for the hot stimulus, 88.9% ± 16.4 % for the cold stimulus and 100% ± 0 % for the mechanical stimulus. These results highlight the ability of all the subjects to correctly recognize the mechanical stimulus. Moreover, an inter-variability that increases with the cold and the hot stimulus can be observed. On the forearm, the results of the correct responses are: 75% ± 15% for the hot stimulus, 97.2% ± 9.6% for the cold stimulus and 94.4% ± 12.9% for the mechanical stimulus. The inter-variability was quite lower on the forearm when compared to the hand, except for the mechanical stimulus. Lastly, total results on the hand and on the forearm are the following: 70.8% ± 21.5% for the hot stimulus, 93.1% ± 11.1 % for the cold stimulus and 97.2% ± 6.5% for the mechanical stimulus. The inter-variability is found to be generally quite low. Furthermore, it is lower for the mechanical stimulus and increases with cold and hot stimuli.

## Discussion

The results of MIT analysis show a higher value of pressure needed to identify a sensation on the forearm than on the hand. This is probably due to the higher density of mechanoreceptors on the hand with respect to the forearm^37^. Moreover, an additional explanation can lie in the fact that the glabrous skin (fingers, palmar surface of the hands, soles of the feet and lips) is more sensitive to touch stimuli than hairy skin^38^. The obtained MIT for the hand and the forearm are not directly compared with the analyzed literature, since two substantially different methods were used to collect data: in this study, a mechanical continuous stimulus was applied with a fixed area whereas in the literature a Von Frey filament was employed, which delivers a discrete stimulus with a different stimulation area for each desired force^1,3,6,8^. The quality of the sensation perceived by the participants may be affected by the use of different contact areas^19^, resulting in uneven values among the literature results. Thus, the use of the developed testbed allows the application of the desired force automatically and with a fixed contact area, enhancing the repeatability of the performed test.

The obtained MPT values are coherent with the analyzed literature and no statistically significant difference was found when comparing the hand and the forearm. More in detail, our MPT value on the hand is higher than in literature data^4,5,9^. A more extensive comparison is depicted in Table 1. These results can be justified by the use of a larger stimulation area ($0.28-1$ cm$\phantom{0}^2$) in the literature compared with the one used in this paper (0.005 cm$\phantom{0}^2$). This explanation is fully supported by^19,20^, which investigated the influence of the stimulation area on the painful threshold on the forearm. Indeed, it was demonstrated that a smaller stimulation area needs a higher pressure to induce a pain sensation. Table 1 shows a direct comparison with these studies. Despite the use of different technologies (Von Frey filament and mechatronic testbed), the obtained results are consistent with the literature, demonstrating the capability of the developed testbed to investigate mechanical thresholds. A statistically significant difference was found between the MIT and MPT on both the hand and forearm, highlighting the capability to deliver distinguishable stimuli between an innocuous and painful stimulus.

The THPT results are almost consistent with the literature on both the analyzed anatomical sites. More in detail, a direct comparison with the literature is reported in Table 1. The mean THPT on the hand ($49.0~^\circ$C) is slightly higher than the values extracted from the literature^6,12,13,15^, even though it falls within the range of the minimum value ($41.6~^\circ$C) found in^15^ and the maximum value ($48.99~^\circ$C) found by^12^. Whereas the THPT result on the forearm ($43.9~^\circ$C) is slightly lower than the literature findings ($46.0~^\circ$C, $47.0~^\circ$C and $44.4~^\circ$C respectively in^1,6,29^). However, both the values of THPT on the hand and on the forearm are in a wider range compared to the literature. This is probably due to the use of a smaller stimulation tip in the present study, which could have made it more difficult to identify the exact temperature associated with pain.

Moreover, THPT on the hand was found to be significantly higher than on the forearm. This can be due to the nature of the stimulated skin. It was previously demonstrated that glabrous skin presents a lower heat pain threshold compared to hairy one, probably because glabrous skin is thicker, *i.e.*with more epidermal layers, and may then affect the rate of heat transfer to the nociceptors and induce a higher time latency (0.7–1.1 s on fingertip compared to 0.4–1.1s on the arm)^32,34^.

Moreover, the identified TWST on the hand is almost $10~^\circ$C higher when compared with studies^6,12,13,15^, as visible from Table 1. Even on the forearm the TWST is $4-7~^\circ$C higher than the data extracted from^1,6,16^, as shown in Table 1. The TCST on both the hand and the forearm are lower than those found in the literature. More in detail, the TCST on the hand is almost $17~^\circ$C lower than studies in^6,12,13,15^, and the TCST on the forearm is almost $10~^\circ$C lower than results from^1,6,16^, as shown in Table 1. This discrepancy is probably due to the use of a smaller stimulation tip (0.07 cm$\phantom{0}^2$) compared to the typical values in the literature ($5-24~$cm$\phantom{0}^2$). A smaller tip is more difficult to be recognized since it covers a reduced area of thermal receptors^30^, leading to a higher TWST and a lower TCST. However, the response to a focused stimulus was namely investigated as it has been purely studied so far. Furthermore, we found that the variability in the perceived temperature is higher for stimuli closer to the skin temperature, as also observed in^31^. TCST on the hand was found to be significantly lower than on the forearm. The experimental results for the TCPT on the hand ($7.9~^\circ$C) and on the forearm ($9.5~^\circ$C) are comparable with those found in the literature ($8-11~^\circ$C on the hand^6,12,13,15^ and $5-10~^\circ$C on the forearm^1,6^).

Additionally, a statistically significant difference was observed between the TCST and TCPT on both the hand and the forearm. This difference is not observable between the TWST and THPT. It can be due to the lower discrepancy in temperature between the skin and a warm temperature compared to a cool one^23^. Additionally, in^31^it was observed that cool and warm stimuli activate different receptors, resulting in a stronger response to a warm stimulus with respect to cool ones, even if the applied temperature variation is the same. Furthermore, 3 participants experienced difficulty in discerning between thermal sensation and thermal pain. They perceived a hot pain soon after the identification of the warm sensation threshold. This finding aligns with the hypothesis proposed in^30^, which suggests that the pain receptors can be likely involved instead of the warm receptors.

The ability to accurately identify the type of painful stimulus was found to be the focus of only one study^23^ in which a fixed temperature was used as a painful stimulus for all the subjects. As highlighted in the results of the previous analysis, there is a high inter-variability in the identified painful thresholds. Hence, to reduce the influence of the applied temperature value on the final response, the subjective THPT and TCPT identified in the previous experimental phase were applied as painful stimuli to each subject. Our analysis, in accordance with that study, demonstrated how the mechanical stimulus presents a higher recognition rate than the hot and cold stimuli. The use of a different stimulation tip between the mechanical and thermal stimulation, despite the final contact area is the same, could be responsible for the higher recognition rate for the mechanical stimulus compared with the hot and cold stimuli. Moreover, the cold stimulus is sometimes confused with the hot one, but never with the mechanical one, maybe due to the activation of only $A\delta$ *mechanical nociceptors* which do not mediate for cold pain but only for mechanical and hot pain. On the other hand, the hot painful stimulus, that was confused with both the hot and the cold stimuli, probably activates the *C* fibers. These fibers are mainly composed of* polymodal nociceptors*, which can respond to various stimuli, including painful mechanical, painful hot, and painful cold^38^. Moreover, the analysis of the standard deviation over the ability to correctly recognize the painful stimulus revealed a low inter-variability, particularly in the recognition of mechanical stimuli. The use of the proposed testbed in this analysis can significantly improve the accuracy and repeatability of the experimental procedure if compared with^23^, since we automatically applied the stimuli in a single experimental section, by using a mechanical and a thermal stimulation tip with the same dimension. Furthermore, the reliability of the study can be enhanced thanks to the use of a single device that includes both the mechanical and thermal tips with the same ending area.

When compared with the state-of-the-art technologies, the developed mechatronic testbed enables: *i)* a high spatial resolution (0.5 $\mu$m) and repeatability thanks to the use of the automated cartesian positioning system as well as the custom restrainer system used to fix the hand/arm position in contrast with the manual application of the stimulus usually employed in literature; *ii)* the application of a mechanical and thermal continuous stimulus, that increases its intensity by maintaining the same contact area during all the stimulation process; *iii)* a constant application velocity, which is limited only by the step response time of the mechanical stimulator (2 ms).

## Conclusions

This study proposes a novel testbed that includes both a mechanical and a thermal stimulator in a single device. This makes it possible to combine mechanical and thermal stimulation within a single experimental session. For instance, it easily allows for the exploration of the human ability to recognize the nature of a painful stimulus that has only been poorly investigated so far. In this work the mechanical and thermal stimuli were not delivered simultaneously. However, the developed testbed can potentially be used with this aim, with the only current limitation consisting of stimulating two different sites with a fixed distance of 8 cm. A different distance can be potentially achieved by a proper adjustment of the stimulators location. Compared with state-of-the-art solutions, the presented testbed allows automated mechanical and thermal stimulation, overcoming the disadvantages associated with the manual application of stimuli. Furthermore, mechanical stimulation presents a fixed stimulation area and delivers a continuous force stimulus, avoiding any influence on the participant’s judgment. It also allows the desired anatomical area to be stimulated in a repeatable manner, thanks to the integration of a high-resolution positioning system. Moreover, the use of a single device embedding both mechanical and thermal stimulators ensures the uniformity of the two stimulation procedures in terms of spatial resolution and velocity of application. This solution can also speed up the experimental process, since there is no need to change instrumentation and setup between two types of stimulation. Moreover, it enables the alternating application of mechanical and thermal stimuli.

The performance of the testbed was tested by exploring the mechanical and thermal thresholds in the human hand and forearm. The obtained results for the mechanical threshold identification are consistent with the data extracted from the literature. MIT results demonstrated the difference between our continuously increasing force delivery and the discrete stimulations pursued with standard devices. MPT results were found to be consistent with the literature, also confirming the dependency of the values from the dimension of the stimulation surface. No statistically significant difference was observed when comparing the results from the two stimulated anatomical sites. A statistical difference between the identified MIT and MPT, regardless of the tested anatomical spot, was found, highlighting the capability of the testbed to produce a range of forces that can induce different sensations (touch or pain).

The obtained results for the thermal pain thresholds are consistent with the data extracted from the literature. Whereas, the thermal sensation thresholds differ from the literature due to the smaller stimulation area used in the presented testbed. The used stimulation tip was appositely selected to study the reaction to a focused thermal stimulus that has been poorly investigated so far.

Lastly, the use of the testbed to recognize the nature of painful stimuli was investigated, and the obtained results are consistent with the available literature. The developed testbed can speed up the experimental process, since the mechanical and thermal stimulators are integrated into a single device that includes a high-resolution positioning system. Moreover, the accuracy of the the applied force and temperature is increased as well as the repeatability and consistency. Furthermore, since the used tip for the mechanical and thermal stimulation presents the same contact area, the testbed allows for a more fair comparison among the applied stimuli.

Future work will be devoted to analyze a larger sample of subjects with the objective of enhancing the consistency of the study. Moreover, thanks to its versatility, the testbed will be used, in the framework of the European project SOMA^39^, to conduct also *in-vitro* tests on an innervated skin model to record its electrical response.

## Data Availability

The datasets used and/or analyzed during the current study are not openly accessible but are available from the corresponding author on reasonable request.
